# Correction: Mitochondrial Dysfunction Promotes Breast Cancer Cell Migration and Invasion through HIF1α Accumulation via Increased Production of Reactive Oxygen Species

**DOI:** 10.1371/journal.pone.0114346

**Published:** 2014-11-25

**Authors:** The PLOS ONE Staff

Figures 3 and 6 are incorrect. The authors have provided a corrected version of each figure here.

**Figure 3:**
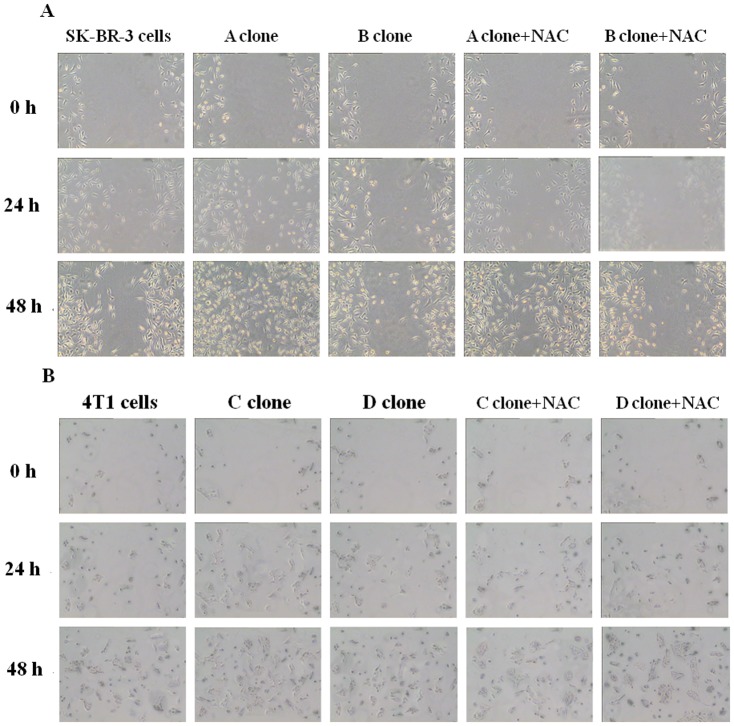
The migration capacity of subclones and their parental cells was measured by wound healing assay. A–B, The subclones migrated faster than the parental cells SKBR3 cells (A) and 4T1 cells (B). NAC was able to effectively inhibit the migration of the subclones.

**Figure 6:**
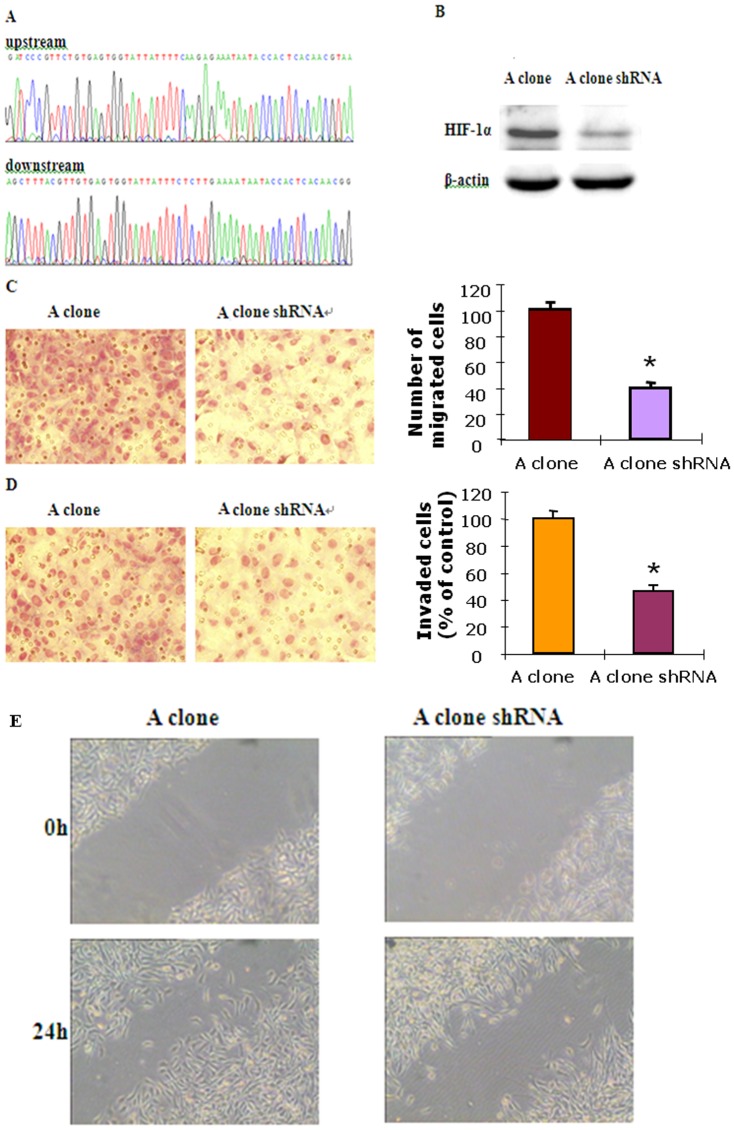
ROS promoted aggressive cellular behaviors by up-regulating HIF-1α expression. A, The sequence of inserts in the shRNA vector clone. B, Cells with HIF-1α shRNA showed decreased HIF-1α expression compared with the control. C, Cells with HIF-1α shRNA showed decreased migration ability compared with the control as documented by transwell migration assay. **P<0.05 vs A clone (n  =  3) D, Cells with HIF-1α shRNA showed decreased invasive capacity compared with the control as documented by transwell invasion assay. **P<0.05 vs A clone (n  =  3) E, Wound healing assay showed that cells with HIF-1α shRNA migrated slower than the control.
